# The taxonomic accounts of the genus *Symmorphus* Wesmael (Hymenoptera, Vespidae, Eumeninae) from China, with descriptions of three new species

**DOI:** 10.3897/zookeys.389.7045

**Published:** 2014-03-14

**Authors:** Ting-jing Li, Bin Chen

**Affiliations:** 1 Institute of Entomology & Molecular Biology, College of Life Sciences, Chongqing Normal University, Chongqing 401331, China

**Keywords:** Hymenoptera, Vespidae, Eumeninae, *Symmorphus*, new species, China

## Abstract

In this study, we recognize and review a total of 19 species of the genus *Symmorphus* Wesmael from China. We also provide a key to these species. Three new species are described and illustrated, namely *Symmorphus (Symmorphus) tianchiensis* Li & Chen, **sp. n.**, *S. (S.) cavatus* Li & Chen, **sp. n.**, and *S. (S.) nigriclypeus* Li & Chen, **sp. n.** The following four species are newly recorded from China: *Symmorphus (S.) fuscipes* (Herrich-Schaeffer), *S. (S.) lucens* (Kostylev), *S. (S.) sublaevis* Kostylev, and *S. (S.) violaceipennis* Giordani Soika. In addition, we map the species geographical distributions in China of these 19 species. Type specimens of these three new species are deposited in Chongqing Normal University and Yunnan Agricultural University.

## Introduction

The genus *Symmorphus* Wesmael contains 44 species with two subspecies, and is distributed in the Palearctic, Oriental, Nearctic regions and the northernmost Neotropical region. These species are usually slender and easily recognized by the combination of the following characters: mesoscutum with well-developed notaulices; metasomal tergum 1 distinctly narrower than tergum 2, but not petiolate, with a basal transverse carina and a median longitudinal furrow; and antennal apex in male simple, not forming a recurved hook. The known species of the genus were described or revised in detail by Giordani Soika (1975), Tsuneki (1977), Cumming and van der Vecht (1986), Cumming (1989), Yamane (1990), Gusenleitner (1999, 2000, 2002, 2004, 2010), Kim and Lee (2002, 2006), and so on. However, a systematic research on the Chinese *Symmorphus* is absent. Twelve species were already recorded from China (Giordani Soika 1966, 1986; Li 1981, 1985; Cumming 1989; Gusenleitner 1999, 2000, 2002, 2004). During the study of the Chinese eumenine wasps, 19 species of *Symmorphus* are recognized, including three new species and four new records. In the present paper, a key to all Chinese species of *Symmorphus* is updated and the species geographical distributions in China are mapped (Fig. 22). In addition, we also provide the taxonomic information and global distributions of these species. The key and distributions were produced based on both the examination of specimens and the information extracted from literatures.

## Materials and methods

The specimens examined are deposited in the Institute of Entomology and Molecular Biology, Chongqing Normal University, Chongqing, China (CQNU) and Department of Entomology, College of Plant Protection, Yunnan Agricultural University, Kunming (YNAU), respectively. Descriptions and measurements were made under a stereomicroscope (Nikon SMZ1500), and all figures were taken with a stereomicroscope (LEICA EZ4HD) attached to a computer using Leica Application Suite version 2.1.0 software. The ratios used throughout the descriptions were measured in the same amplifying multiple of stereomicroscope. All measurements were taken as the maximal length of body parts measured. Body length was measured from the anterior margin of head to the posterior margin of metasomal tergum 2. For the density description of punctures, the phrase widely spaced means that the intervals between are larger than diameter, moderately spaced means equal to diameter, and whereas closedly spaced means less than diameter. The abbreviations used in the text are shown as follows: T1 for metasomal tergum 1, T2 for metasomal tergum 2, S1 for metasomal sternum 1, S2 for metasomal sternum 2, and so on. Terminology principally follows Carpenter (1982) and Cumming and van der Vecht (1986).

## Taxonomy

### Symmorphus

Wesmael, 1836

Symmorphus Wesmael, 1836: 45, subgenus of *Odynerus* Latreille; Li 1985: 115; Cumming 1989: 13–15; Kim and Lee 2006: 27–28.

#### Type species.

*Odynerus elegans* Wesmael, 1833, designated by Richards (1935).

### Symmorphus (Symmorphus) tianchiensis

Li & Chen sp. n.

http://zoobank.org/FCA23B0A-F2DB-499B-B1B6-15D75C34CBAB

http://species-id.net/wiki/Symmorphus_tianchiensis

Figs 1–10

#### Material examined.

Holotype. ♀, China, Yunnan Prov., Dali City, Yunlong County, Tianchi National Nature Reserve, 25°52'13.05"N, 99°17'14.33"E, 2579 m, 9.VII.2011, Tingjing Li, No. 1001516 (CQNU); **Paratypes:** 1♀1♂, the same as holotype, Nos. 1001524, 1001517 (CQNU); 1♀, China, Yunnan Prov., Nujiang City, Lanping County, Jinding Town, Xinshengqiao National Forest Park, 26°26'56.36"N, 99°23'04.69"E, 2412 m, 12. VII.2011, Tingjing Li, No. 1004036 (CQNU).

#### Description.

**Figures 1–10.**
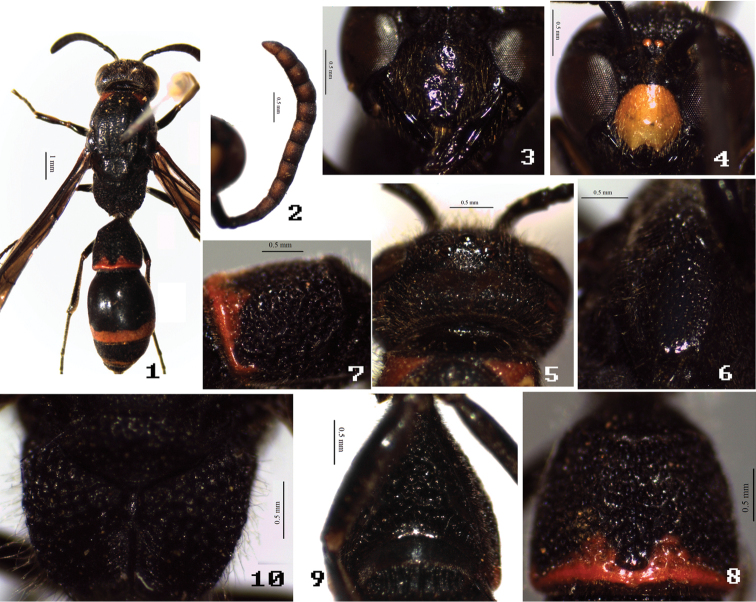
*Symmorphus (Symmorphus) tianchiensis* Li & Chen, sp. n. **1, 3, 5–10** female, **2, 4** male. **1** general habitus **2** antenna in ventral view **3–4** clypeus **5** vertex in dorsal view **6** mesepisternum in ventral view **7** transverse carina of T1 in lateral view; **8** T1; **9** S1; **10** propodeum.

Female (Fig. 1): body length 9.5–10.5 mm, forewing length 10.0–10.5 mm. Black; with the following parts orange-red to red: dorsal pronotal spot, apical border of T1, and subapical border of T2, S2 and T4 (absent in one female paratype); interantennal spot and post-ocular dot orange-yellow; fore tibia inside brown. Wings lightly infuscate. Hairs pale brown; mesosoma with sparse lengthened hairs, in addition to short pubescence.

Head. Clypeus sparsely punctate-puncticulate to foveolate-puncticulate, apex broadly and moderately emarginated, evenly convex, with apical teeth, and slightly reflexed anteriorly (Fig. 3); frons and vertex foveolate-puncticulate, major punctures closely spaced; interantennal carina prominent; cephalic fovea somewhat larger than post ocellus, and foveal interval somewhat less than postocellar distance, cephalic foveal carina indistinct (Fig. 5); occipital carina without submedial incisions.

Mesosoma. Pronotum, except anterior face, foveolate-puncticulate, major punctures closely spaced, more or less costate laterally, anterior face smooth and polish, pronotal carina complete, humeral angle barely projected. Mesoscutum foveolate-puncticulate, major punctures widely spaced medially, moderately spaced anteriorly and posteriorly; notaulus complete; mesepisternum with epicnemial carina dorsally obsolete and ventrally faint (Fig. 6); anterior margin of pseudosternum without high reflexed margin; mesoscutellum foveolate-puncticulate, similar to those on mesoscutum. Dorsal mesepisternum sparsely foveolate-puncticulate, space between punctures smooth and polish, ventral mesepisternum sparsely foveolate-puncticulate, major punctures widely spaced, minor punctures obscure, space between punctures alutaceous; dorsal mesepimeron sparsely foveolate, space between punctures smooth and polish, ventral mesepimeron dull, and coarsely alutaceous. Metanotum foveolate-puncticulate dorsally, obscurely puncticulate ventrally, metanotum nearly vertical, dorsal surface narrow. Propodeal dorsum and posterior face coarse, areolate-rugose, propodeal superior shelf moderately long (2.2 times trans-scutal sulcus), lateral face of propodeum striately rugose ventrally, areolate-rugose dorsally, propodeal submedian carina present ventrally, faint and irregular dorsally (Fig. 10), propodeal valvula short posteriorly, fused distally to submarginal carina, propodeal orifice somewhat elliptic dorsally (Fig. 10).

Metasoma. Metasomal tergum 1 with postcarinal area foveolate-puncticulate to foveate-puncticulate (Figs 7–8), postcarinal length of T1/apical width=0.73, carinal width/apical width=0.79, postcarinal sides slightly convergent toward base, transverse carina laterally faint (Fig. 7), longitudinal furrow narrowly and shallowly depressed, with deeper narrow medial sulcus (Fig. 8), apical margin indistinctly depressed; S1 flat and without basal carina anteriorly, areolate-rugose medially and posteriorly, without median longitudinal ridge, and lateral oblique ridges strongly prominent (Fig. 9); segment 2 foveolate-puncticulate basally, major punctures more closely spaced; T3–5 sparsely punctate-puncticulate to foveolate-puncticulate, major punctures subapically; segment 2 except base and S3–6 with evenly minor punctures.

Male. Body length 7.5 mm, forewing length 8.5 mm. Sculpture, punctuation, hairs, and coloration similar to female except as follows: clypeus entirely light yellow to yellow (Fig. 4), except apical margin brown red; antennal segments 3–13 pale brown ventrally (Fig. 2); dorsal pronotal spot, apical border of T1, and subapical border of both T2 and S2 orange-red; mesepisternum dorsally with small yellow spot; fore tibia inside pale brown, fore tarsus brown apically; antennal tyloids absent, segment 13 length/width=1.56 (Fig. 2).

#### Distribution.

China (Yunnan).

#### Remarks.

This species is similar to *Symmorphus (Symmorphus) sichuanensis* by S1 without basal carina, T1 with transverse carina laterally faint to obsolete, and mesepisternum with epicnemial carina dorsally obsolete; but can be easily distinguished from the similar species and other members of the genus by the combination of the following characters: body moderately long; in both female and male, dorsal pronotal spot orange-red (Figs 1, 5); in female, mesepisternum with epicnemial carina ventrally faint; in male, antenna without tyloids (Fig. 2), and subapical border of both T2 and S2 orange-red.

#### Etymology.

It is named after the type locality of the species, Tianchi National Nature Reserve in Dali city, Yunnan of China.

### Symmorphus (Symmorphus) cavatus

Li & Chen sp. n.

http://zoobank.org/AA3DF78F-D616-4B15-9E0E-8E5FE65CAD35

http://species-id.net/wiki/Symmorphus_cavatus

Figs 11–16

#### Material examined.

Holotype. ♀, China, Yunnan Prov., Xishuangbanna, Jinghong City, Jinuo mountain, 22°02'17.81"N, 101°00'15.36"E, 901 m, 12.IV,2010, Rui Zhang, No. 1004037 (YNAU).

#### Description.

**Figures 11–16.**
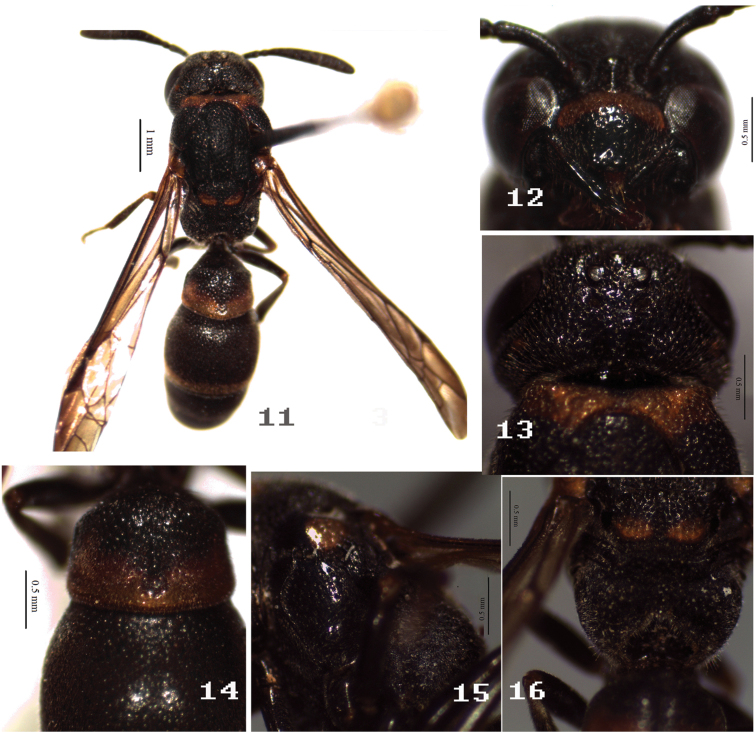
Female of *Symmorphus (Symmorphus) cavatus* Li & Chen, sp. n. **11** general habitus **12** clypeus **13** vertex in dorsal view **14** T1–2 **15** mesepisternum; **16** propodeum.

Female (Fig. 11): body length 6.5 mm, forewing length 7.0 mm. Black; with the following parts pale brown: basal transverse band of clypeus, post-ocular dot, medially uninterrupted dorsal pronotal band, large dorsal mesepisternal spot, tegula, large mesoscutellar spot apically, apex of T1, apical margin of both T2 and S2, apex of fore femur, and fore tibia largely; fore and mid tarsi dark brown. Wings lightly infuscate. Hairs pale brown, mesosoma without sparse lengthened hairs, in addition to short pubescence.

Head. Clypeus moderately punctate-puncticulate to foveolate-puncticulate, punctures sparse medioapically, space between punctures smooth and polish; clypeal apex truncated, clypeus evenly convex, without apical teeth (Fig. 12). Frons and vertex foveolate-puncticulate, major punctures closely spaced; interantennal carina prominent; cephalic fovea somewhat smaller than post ocellus, foveal interval subequal to postocellar distance, without cephalic foveal carina; occipital carina with 2 submedial incisions.

Mesosoma. Pronotum, except anterior face, foveolate-puncticulate, with major punctures closely spaced, more or less costate laterally; anterior face distinctly imbricate; pronotal carina dorsally obsolete; humeral angle slightly projected. Mesoscutum foveolate-puncticulate, major punctures closely spaced anteriorly and posteriorly, widely spaced laterally; notaulus complete; epicnemial carina complete; pseudosternum anterior margin without high reflexed margin. Mesoscutellum foveolate-puncticulate, major punctures closely spaced, with shallowly medial furrow. Mesepisternum with complete epicnemial carina (Fig. 15), dorsally punctate-puncticulate and minor punctures distinct basally, other parts foveolate-puncticulate, major punctures widely spaced, minor punctures obscure. Mesepimeron dull and densely striate. Metanotum primarily oblique and not vertical, dorsally dull, coarse and areolate-rugose. Propodeum dull, densely striate laterally, areolate-rugose dorsally, posterior face deeply heart-shaped hollowed, margin reflexed, complete and sharply defined throughout (Fig. 16), propodeal superior shelf short, 2 times trans-scutal sulcus; propodeal submedian carina entirely absent, propodeal valvula short posteriorly, fused distally to posterior margin; propodeal orifice small and indistinct.

Metasoma. Metasomal tergum 1 with postcarinal area foveolate-puncticulate, major punctures densely spaced, postcarinal length short, postcarinal length/apical width=0.68, carinal width/apical width=0.85, postcarinal sides barely convergent toward base, transverse carina complete, longitudinal furrow narrowly and shallowly depressed, with deeper narrow medial sulcus, apical margin indistinctly depressed (Fig. 14); S1 rugose anteriorly, basal carina inflated and raised posteriorly, fused to lateral oblique ridges, with median longitudinal ridge, lateral oblique ridges slightly prominent, median longitudinal ridge flanked by longitudinal carinate rugae; segment 2 foveolate-puncticulate, major punctures widely spaced from base to apex, minor punctures connected by obscure imbricate subsculpture; T3–T5 and S3 with densely foveolate toward apex; S3–S6 with imbricate subsculpture.

Male. Unknown.

#### Remarks.

This species is easily distinguished at once from all other species of *Symmorphus* by the combination of the following characters: propodeal posterior face deeply hollowed (Fig. 16), and occipital carina with 2 submedial incisions, in other species of the genus propodeal posterior face not hollowed.

#### Distribution.

China (Yunnan).

#### Etymology.

The specific name is the Latin *cavatus* (= hollow), which refers to propodeal posterior face of the species deeply hollowed.

### Symmorphus (Symmorphus) nigriclypeus

Li & Chen sp. n.

http://zoobank.org/C5EFD701-81FE-44EA-B533-E1531BA283FD

http://species-id.net/wiki/Symmorphus_nigriclypeus

Figs 17–21

#### Material examined.

Holotype. ♂, China, Tibet, Nyingchi, Medog County, 29°71'N, 95°63'E, 3026 m, 13.VII.2013, Yong Zhou, No. 1004038 (CQNU).

#### Description.

**Figures 17–21.**
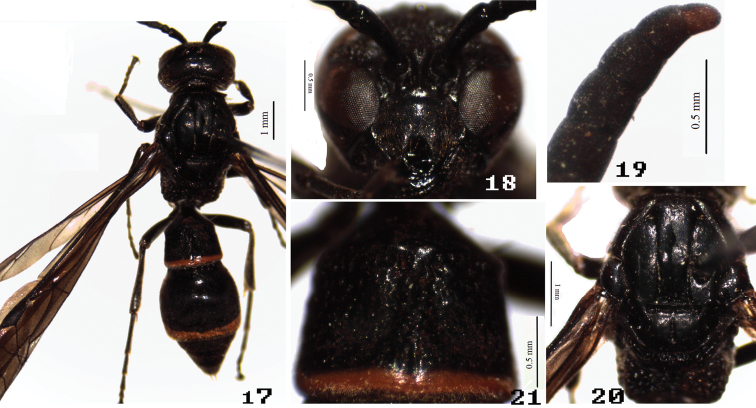
Male of *Symmorphus (Symmorphus) nigriclypeus* Li & Chen, sp. n. **17** general habitus **18** clypeus **19** antennal apex in lateral view **20** mesosoma in dorsal view **21** T1.

Male (Fig. 17): body length 7.0 mm, forewing length 9.0 mm. Black; apical margin of T1 orange-red, subapical margin of both T2 and S2 orange-yellow. Wings lightly infuscate. Hairs white, mesosoma with sparse lengthened hairs, in addition to short pubescence.

Head. Clypeus moderately punctate-puncticulate, clypeal apex deeply emarginated and with acute apical toothed laterally (Fig. 18). Frons punctate-puncticulate to foveolate-puncticulate, major punctures closely spaced; vertex punctate-puncticulate and barely foveolate-puncticulate, minor punctures closely spaced; interantennal carina prominent; male antennal segments 10–13 with tyloids, segment 13 length/width=1.43 (Fig. 19).

Mesosoma. Pronotum, except anterior face, punctate-puncticulate to foveolate-puncticulate, major punctures widely spaced, more or less costate laterally, minor punctures evenly and closely spaced; anterior face obscurely imbricate; pronotal carina complete; humeral angle moderately projected. Mesoscutum punctate-puncticulate to foveolate-puncticulate, primarily punctate-puncticulate, minor punctures evenly and densely spaced, major punctures great widely spaced; notaulus complete and distinctly deep (Fig. 20); pseudosternum anterior margin without high reflexed margin. Mesoscutellum similar to mesoscutum. Dorsal mesepisternum sparsely punctate-puncticulate to foveolate-puncticulate, major punctures very widely spaced, shallow and indistinct, minor punctures obscure; ventral mesepisternum foveolate-puncticulate, major punctures widely spaced, minor punctures obscure. Mesepimeron striately rugose. Metanotum nearly vertical, dorsal surface narrow, foveolate-puncticulate dorsally, striately rugose ventrally. Propodeum striately rugose laterally, areolate-rugose dorsally; posterior face obscure striately rugose, propodeal superior shelf length 3.1 timestrans-scutal sulcus, propodeal orifice broadly and rounded, propodeal submedian carina complete and sharply defined throughout; propodeal valvula short posteriorly, fused distally to submarginal carina.

Metasoma. Metasomal tergum 1 with postcarinal area foveolate-puncticulate, postcarinal length/apical width=0.71, carinal width/apical width=0.85, postcarinal sides slightly convergent toward base, transverse carina complete, longitudinal furrow broadly depressed, with deeper narrow medial sulcus; apical margin indistinctly depressed (Fig. 21); S1 rugose anteriorly, basal carina deeply curved posteriorly, fused to lateral oblique ridges, median longitudinal ridge strongly prominent and flanked by longitudinal carinate rugae; segment 2 punctate-puncticulate, minor punctures connected by obscure imbricate subsculpture; T3–5 sparsely foveolate-puncticulate, major punctures subapically. Segments 3–6 with imbricate subsculpture.

Female. Unknown.

#### Remarks.

This species is easily distinguished from all other species of *Symmorphus* by the combination of the following characters: body black, except apical margin of T1, and subapical margins of both T2 and S2 (Fig. 17); clypeal apex deeply emarginated and with acute apical toothed laterally (Fig. 18).

#### Distribution.

China (Tibet).

#### Etymology.

The specific name *nigriclypeus* is the Latin *nigr* (= black) + *clypeus* (=clypeus), which refers to the clypeus in male of the species black.

### Symmorphus (Symmorphus) ambotretus

Cumming, 1989

http://species-id.net/wiki/Symmorphus_ambotretus

Symmorphus ambotretus Cumming 1989: 28; Kim and Lee 2002: 284 (key), 286, figs 1–6; 2006: 28, 29 (key).

#### Material examined.

1♀, China, Yunnan Prov., Dali, North Gucheng, 9.V.2007, Rui Zhang; 1♀, China, Yunnan Prov., Dali, Yunlong, Luodun Town, 10.VII.2011, Tingjing Li; 1♀, China, Yunnan Prov., Nujiang, Lanping, Yingpan Town, 13.VII.2011, Zhenhu Wu; 1♀, China, Yunnan Prov., Lijiang, Ninglang, Daxing Town, 25.VII.2011, Tingjing Li; 6♂♂, China, Yunnan Prov., Dehong, Yingjiang, Tongbiguan Natural reserve, 3.V.2013; 1♀, China, Chongqing, Wansheng, Heishangu, 4.V.2011, Zhenhu Wu.

#### Distribution.

China (Sichuan, Yunnan, Chongqing); Nepal; Korea.

### Symmorphus (Symmorphus) angustatus

(Zetterstedt, 1838)

http://species-id.net/wiki/Symmorphus_angustatus

Odynerus angustatus Zetterstedt, 1838: 457.
Odynerus suecicus de Saussure, 1855: 187 (key), 190, pl. X fig. 3.
Odynerus laeviventris Thomson, 1874: 86; Gussakovskii 1932: 55.
Symmorphus angustatus (Zetterstedt): van der Vecht 1971: 127; van der Vecht and Fischer 1972: 119 (cat.); Cumming 1989: 3, 5, 23 (key), 44; Kurzenko 1995: 318; Kim and Lee 2002: 285 (key), 290, figs 33-40; 2006: 28 (key), 35-36; Gusenleitner 2003: 864; Castro and Dvořák 2009: 299.
Symmorphus hakutozanus Tsuneki, 1986: 23–24, 26; Cumming 1989: 44.
Symmorphus nansetsurei Tsuneki, 1986: 26, 27; Cumming 1989: 44.
Symmorphus iwatai Yamane, 1990: 115 (key), 127–128, synonymized by Kurzenko 1995.

#### Material examined.

1♀, China, Jilin Prov., Tonghua, Mehekou, 22.VIII.1993, Zhihong Li.

#### Distribution.

China (Jilin); Norway; Sweden; Finland; Denmark; France; Germany; Austria; Greece; Turkey; Belarus; Russia (to Primorskij Krai); Kazahkstan; Mongolia; the Korean Peninsula; Japan.

### Symmorphus (Symmorphus) apiciornatus

(Cameron, 1911)

http://species-id.net/wiki/Symmorphus_apiciornatus

Ancistrocerus apiciornatus Cameron, 1911: 288.
Odynerus (Ancistrocerus) apiciornatus (Cameron): von Schulthess 1934: 74; Giordani Soika 1941: 232.
Symmorphus apiciornatus (Cameron): van der Vecht and Fischer 1972: 119; Giordani Soika 1975: 150, 156, figs 4, 9; Tsuneki 1977: 15; Giordani Soika 1986: 153; Cumming 1989: 3, 5, 24 (key), 79; Kurzenko 1995: 316; Kim and Lee 2002: 286 (key), 295, figs 70-78; 2006: 29 (key), 38-40.
Symmorphus seoulensis Tsuneki, 1986: 22, figs 66-67, female; Cumming 1989: 5, 79.

#### Material examined.

1♀, China, Beijing, Haidian, 29.IV.1952. Jikun Yang; 1♀, China, Beijing, Gongzhufen, 12.V.1952. Jikun Yang; 1♂, China, Shaanxi Prov., Baoji, Fengxian, Jialing River, 26.V.2007, Xiaoyu, Jiang.

#### Distribution.

China (Beijing, Shaanxi, Fujian, Guangdong, Jiangsu, Sichuan); Russia; South Korea; Japan.

### Symmorphus (Symmorphus) aurantiopictus

Giordani Soika, 1986

http://species-id.net/wiki/Symmorphus_aurantiopictus

Symmorphus aurantiopictus Giordani Soika, 1986: 154, fig. 47; Cumming 1989: 3, 5, 22 (key), 81.

#### Material examined.

No specimens examined.

#### Distribution.

China (Jiangsu).

### Symmorphus (Symmorphus) bifasciatus

(Linnaeus, 1761)

http://species-id.net/wiki/Symmorphus_bifasciatus

Vespa bifasciata Linnaeus, 1761: 419; Richards 1935: 163.
Symmorphus bifasciatus (Linnaeus): van der Vecht and Fischer 1972: 119 (cat.); Li 1985: 115 (key), 117; Cumming 1989: 3, 5, 24 (key), 57; Kurzenko 1995: 315; Kim and Lee 2002: 285 (key), 292, figs 48–54; 2006: 28 (key), 37-38.
Vespa sinuata Fabricius, 1793: 270; Cumming 1989: 5, 57.
Odynerus sinuatus var. *mutinensis* Baldini, 1894: 78, pl. III fig. 6; Cumming 1989: 5, 57.
Symmorphus sparsus Morawitz, 1895: 490; Cumming 1989: 5, 57.
Symmorphus sinuatissimus Richards, 1935: 162; Giordani Soika 1963: 123; Cumming 1989: 5, 57.
Symmorphus mutinensis Baldamus: van der Vecht and Fischer 1972: 122 (cat.); Cumming 1989: 5, 57.
Symmorphus mutinensis auster Giordani Soika, 1975: 150, 160; Cumming 1989: 5, 57.
Symmorphus mutinensis yezoanus Tsuneki, 1977: 16; Cumming 1989: 5, 57.

#### Material examined.

1♂, China, Chongqing, Jiangjin, Simianshan National Nature Reserve, 6.V.2012, Ju You.

#### Distribution.

China (Jiangsu, Chongqing); Europe, eastward to northeastern Siberia and Far Eastern Russia; Kyrgyzstan; Kazahkstan; Korea; Japan.

### Symmorphus (Symmorphus) foveolatus

Gussakovskii, 1932

http://species-id.net/wiki/Symmorphus_foveolatus

**Figures 22.**
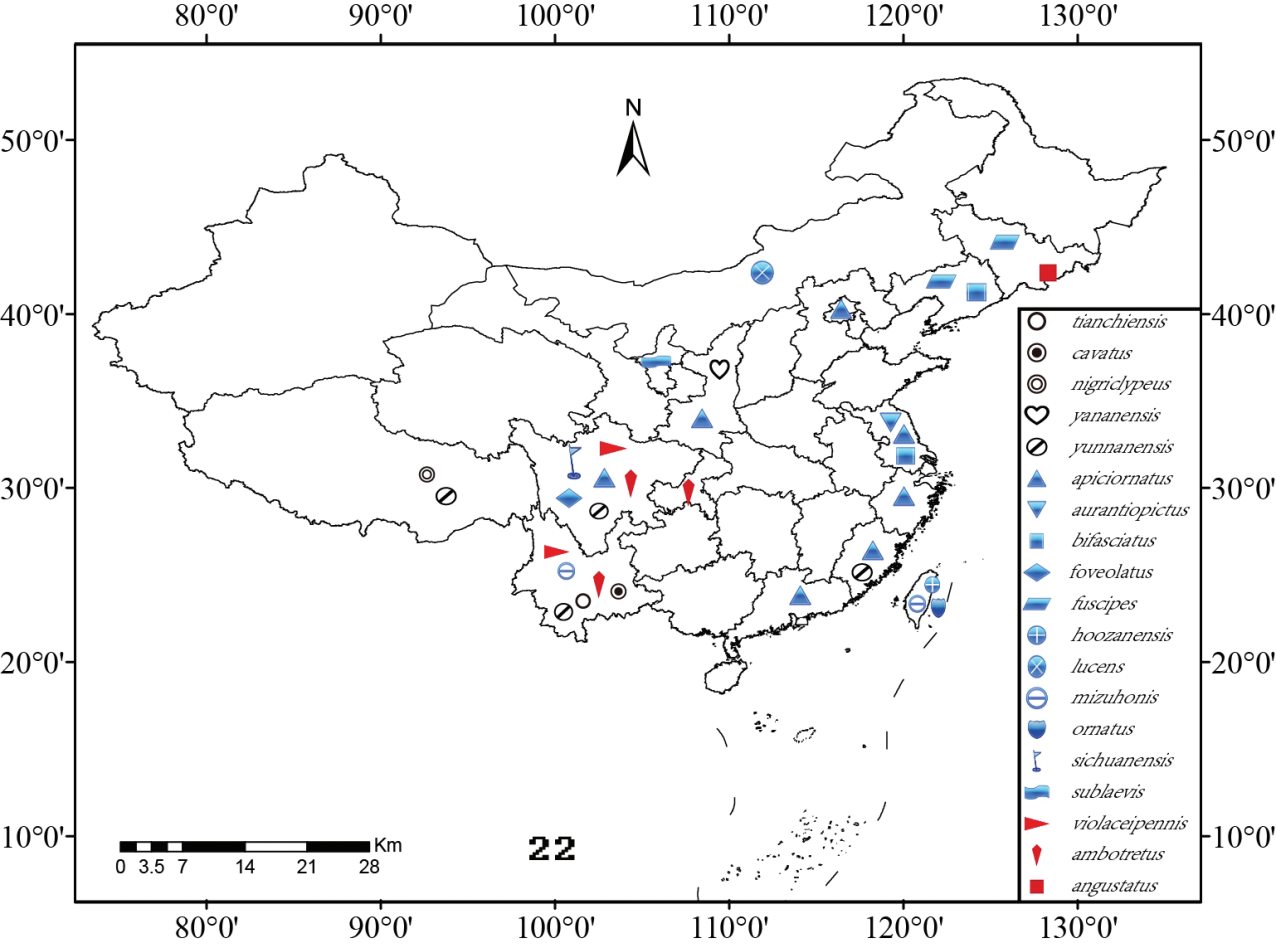
The species geographical distributions of the genus *Symmorphus* in China.

Symmorphus foveolatus Gussakovskii, 1932: 55; van der Vecht and Fischer 1972: 121 (cat.); Tsuneki 1986: 27; Li, 1985: 115–116; Cumming 1989: 3, 5, 23 (key), 35; Kurzenko 1995: 317; Kim and Lee 2002: 285 (key), 289, figs 19–24; 2006: 28 (key), 37–38.
Odynerus captivus Smith: von Schulthess 1934: 66 (key); Yasumatsu 1938: 111, pl. 3 figs 1–5; Kim 1970: 554; 1980: 116. Misidentification.

#### Material examined.

6♀♀2♂♂, China, Sichuan Prov., Panzhihua, Renhe Town, 28.VII.2011, Tingjing Li.

#### Distribution.

China (Sichuan); Russia; the Korean Peninsula; Japan.

### Symmorphus (Symmorphus) fuscipes

(Herrich-Schaeffer, 1838) new record

http://species-id.net/wiki/Symmorphus_fuscipes

Odynerus fuscipes Herrich-Schaeffer, 1838: 18, pl. 18; Cumming 1989: 77 (designation of neotype).
Symmorphus karelicus Morawitz, 1895: 490.
Symmorphus fuscipes (Herrich-Schaeffer): van der Vecht and Fischer 1972: 121 (cat.); Cumming 1989: 3, 5, 22 (key), 77; Kim and Lee 2006: 28 (key), 31.

#### Material examined.

2♀♀, China, Liaoning Prov., Liaoyang, Gongchangling, Anping, 7.VII.2012.Ju You; 1♀, China, Jilin Prov., Changchun, Dehui, Xiajiadian, 28.VI.2012, Ju You; 1♀, China, Jilin Prov., Baishan, Linjiang, Naozhi Town, 7.VII.2012, Ju You.

#### Distribution.

China (new record: Liaoning, Jilin); Norway; Sweden; Finland; Netherlands; Germany; Austria; Belarus; Mongolia; Russia.

### Symmorphus (Symmorphus) hoozanensis

(von Schulthess, 1934)

http://species-id.net/wiki/Symmorphus_hoozanensis

Odynerus hoozanensis von Schulthess, 1934: 67.
Symmorphus hoozanensis (von Schulthess): Cumming, 1989: 3, 5, 21 (key), 26.

#### Material examined.

No specimens examined.

#### Distribution.

China (Taiwan).

### Symmorphus (Symmorphus) lucens

(Kostylev, 1938)

http://species-id.net/wiki/Symmorphus_lucens

Odynerus lucens Kostylev, 1938: 304; Cumming 1989: 66 (designation of lectotype).
Symmorphus lucens (Kostylev): Gussakovskii 1932: 55; van der Vecht and Fischer 1972: 121 (cat.); Cumming 1989: 3, 5, 24 (key), 66; Kim and Lee 2002: 285 (key), 292, figs 41–47; 2006: 29 (key), 36–37.
Symmorphus ishikawai Giordani Soika, 1975: 151, 159; Cumming 1989: 5, 66.

#### Material examined.

2♀♀, China, Inner Mongolia, Helan Mountain, Gulamuxiaosong Hill, 30.VII.2010, Jian Li & Junzhe Xue; 1♀, China, Inner Mongolia, Helan Mountain, Halawu Ravine, 20.VII.2006, Ming Luo; 1♀, China, Inner Mongolia, Helan Mountain, Yushuwan, 27.VII.2010, Fangzhou Ma.

#### Distribution.

China (new record: Inner Mongolia), Russia: southern Siberia to Sakhalin; Korea; Japan.

### Symmorphus (Symmorphus) mizuhonis

Tsuneki, 1977

http://species-id.net/wiki/Symmorphus_mizuhonis

Symmorphus mizuhonis Tsuneki, 1977: 15-20; Cumming 1989: 3, 5, 22 (key), 54; Kurzenko 1995: 316; Kim and Yoon 1996: 205; Kim and Lee 2002: 285 (key), 287, figs 14-18; 2006: 28 (key), 31-32; Castro and Dvořák 2009: 300.
Symmorphus kurentzovi Kurzenko, 1981: 104, figs 111-116; Cumming 1989: 5, 54.
Symmorphus iiyamai Tsuneki, 1986: 26 (key), fig. 70, male; Cumming 1989: 5, 54.
Symmorphus shiroyamai Tsuneki, 1986: 26 (key), 27, fig. 71, male; Cumming 1989: 5, 54.
Symmorphus piceanus Tsuneki, 1986: 26 (key), 27, fig. 72; Cumming 1989: 5, 54.
Symmorphus sassai Tsuneki, 1986: 26 (key), 27, fig. 73; Cumming 1989: 5, 54.

#### Material examined.

2♀♀4♂♂, China, Yunnan Prov., Diqing, Deqin, Near the county, 19.VII.2011, Tingjing Li; 4♀♀, China, Yunnan Prov., Dali, Yunlong, Tianchi, 9.VII.2011, Tingjing Li.

#### Distribution.

China (Yunnan, Sichuan, Taiwan); Russia: Irkutsk, Primorskij Krai; Kazahkstan; North Korea; Japan.

### Symmorphus (Symmorphus) ornatus

Gusenleitner, 2000

http://species-id.net/wiki/Symmorphus_ornatus

Symmorphus ornatus Gusenleitner, 2000: 939, 945.

#### Material examined.

No specimens examined.

#### Distribution.

China (Taiwan).

### Symmorphus (Symmorphus) sichuanensis

Lee, 1981

http://species-id.net/wiki/Symmorphus_sichuanensis

Symmorphus sichuanensis Lee, 1981: 423, fig. 1; Li 1985: 115-116; Cumming 1989: 3, 5, 38.

#### Material examined.

3♀♀: China, Sichuan Prov., Leshan City, Emeishan, Gaoqiao Town, Yanshi Village, 11.VIII.2011, Tingjing Li.

#### Distribution.

China (Sichuan); Thailand.

### Symmorphus (Symmorphus) sublaevis

Kostylev, 1940 new record

http://species-id.net/wiki/Symmorphus_sublaevis

Odynerus sublaevis Kostylev, 1940: 40.
Symmorphus sparsus Morawitz: Giordani Soika 1963: 123. Misidentification.
Symmorphus sublaevis (Kostylev): van der Vecht and Fischer 1972: 122 (cat.); Cumming 1989: 3, 5, 24 (key), 68.

#### Material examined.

5♂♂, China, Ningxia, Jingyuan, Xixia Forest, 15–16.VII.2008, Xiumin Li; 1♂, China, Ningxia, Longde, Sutai Forest, 1–2,VII.2008, Xinpu Wang; 1♂, China, Ningxia, Guyuan, Lvyan Forest, 9–10.VII.2008, Guodong Ren.

#### Distribution.

China (new record: Ningxia); Kyrgyzstan; Kazahkstan.

### Symmorphus (Symmorphus) violaceipennis

Giordani Soika, 1966 new record

http://species-id.net/wiki/Symmorphus_violaceipennis

Symmorphus violaceipennis Giordani Soika, 1966: 102; Cumming 1989: 3, 5, 22 (key), 53.

#### Material examined.

10♀♀, China, Yunnan Prov., Dali, Yunlong, Tianchi, 9.VII.2011, Tingjing Li; 9♀♀, China, Yunnan Prov., Nujiang, Lanping, Yingpan Town, 12.VII.2011, Zhenhu Wu; 1♀, China, Sichuan Prov., Kangding, Paoma Mountain, 7.VII.2005, Hu Zhou.

#### Distribution.

China (new record: Yunnan, Sichuan); India; Nepal.

### Symmorphus (Symmorphus) yananensis

Gusenleitner, 2002

http://species-id.net/wiki/Symmorphus_yananensis

Symmorphus yananensis Gusenleitner, 2002: 345.

#### Material examined.

No specimens examined.

#### Distribution.

China (Shaanxi).

### Symmorphus (Symmorphus) yunnanensis

Gusenleitner, 2002

http://species-id.net/wiki/Symmorphus_yunnanensis

Symmorphus yunnanensis Gusenleitner, 2002: 345; 2004: 1104.

#### Material examined.

5♀♀11♂♂, China, Yunnan Prov., Diqing, Deqin, Near the county, 19.VII.2011, Tingjing Li; 1♂, China, Tibet, Changdu, Mangkang, 3508 m, 5.VII.2013, Yong Zhou.

#### Distribution.

China (Yunnan, Tibet, Fujian).

### Key to the Chinese species of the genus *Symmorphus* Wesmael

**Table:** 

1	Metasomal tergum 2 obtusely angled basally in profile, and S2 abruptly truncate behind basal sulcus	2
–	Metasomal tergum 2 gradually rounded basally in profile, and S2 flat to slightly convex behind basal sulcus	3
2	Pronotal carina complete; occipital carina submedially incised	*Symmorphus (Symmorphus) ambotretus* Cumming
–	Pronotal carina obsolete dorsolaterally, only distinct medially; occipital carina submedially at most depressed, but not incised	*Symmorphus (Symmorphus) hoozanensis* (Schulthess)
3	Mesosoma with sparse lengthened hairs, in addition to dense short hairs	4
–	Mesosoma without sparse lengthened hairs, in addition to dense short hairs	10
4	Metasomal sternum 1 with basal carina	5
–	Metasomal sternum 1 without basal carina	7
5	Body black, except apical margin of T1 and subapical margin of both T2 and S2 (Fig. 17); clypeal apex deeply emarginated (Fig. 18)	*Symmorphus (Symmorphus) nigriclypeus* sp. n.
–	Coloration of body different from the above, or clypeal apex not deeply emarginated	6
6	Propodeum with superior shelf moderately long, medial length 2.50–3.11 times length of trans-scutal sulcus; in female, cephalic fovea very small and its maximum diameter 0.25–0.33 times trans-scutal sulcus length, clypeus deeply emarginated apically and with teeth slightly reflexed anteriorly	*Symmorphus (Symmorphus) violaceipennis* Giordani Soika, new record
–	Propodeum with superior shelf shorter, medial length 1.38–2.21 times length of trans-scutal sulcus; in female, cephalic fovea larger and its maximum diameter greater than 0.50 times trans-scutal sulcus length, clypeus moderately emarginated apically and without reflexed teeth	*Symmorphus (Symmorphus) mizuhonis* Tsuneki
7	Metasomal tergum 1 with complete transverse carina	*Symmorphus (Symmorphus) angustatus* (Zetterstedt)
–	Metasomal tergum 1 with transverse carina laterally faint to obsolete	8
8	Mesepisternum without epicnemial carina	*Symmorphus (Symmorphus) foveolatus* Gussakovskij
–	Mesepisternum with or at least faint epicnemial carina ventrally	9
9	Mesosoma with orange-red maculation (Figs 1, 5); male antenna without tyloids (Fig. 2)	*Symmorphus (Symmorphus) tianchiensis* sp. n.
–	Mesosoma black; male antenna with tyloids	*Symmorphus (Symmorphus) sichuanensis* Lee
10	Pronotal carina obsolete dorsolaterally, only distinct medially; S1 without basal carina	*Symmorphus (Symmorphus) aurantiopictus* Giordani Soika
–	Pronotal carina complete; S1 with basal carina	11
11	Posterior face of propodeum deeply hollowed (Fig. 16); in female, occipital carina with 2 submedial incisions	*Symmorphus (Symmorphus) cavatus* Li & Chen, sp. n.
–	Posterior face of propodeum not hollowed, at most depressed; occipital carina without submedial incisions	12
12	Submedian carina of propodeum strongly developed as a high and sharp complete carina; T1 in postcarinal area distinctly narrowed toward base	*Symmorphus (Symmorphus) fuscipes* (Herrich-Schaeffer), new record
–	Submedian carina of propodeum usually not forming a high and sharp carina; T1 in postcarinal area barely to moderately narrowed toward base	13
13	Lateral and posterior faces of propodeum dull and finely striate	14
–	Lateral face of propodeum not dull, striately to areolately, posterior face shiny, nearly smooth	17
14	Maculation, except male clypeus, red	*Symmorphus (Symmorphus) yunnanensis* Gusenleitner
–	Maculation ivory to yellow	15
15	Metasomal sternum 1 without basal carina	*Symmorphus (Symmorphus) yananensis* Gusenleitner
–	Metasomal sternum 1 with basal carina	16
16	Basal band of clypeus wider in female; punctures on mesonotum, mesopleuren and apex of T2 denser than those of the related species	*Symmorphus (Symmorphus) ornatus* Gusenleitner
–	Basal band of clypeus relatively narrower in female; punctures on mesonotum, mesopleuren and apex of T2 sparser	*Symmorphus (Symmorphus) apiciornatus* (Cameron)
17	Dorsal mesepisternum foveolate-puncticulate, with major punctures large and densely spaced; in male, antennal segment 13 moderately long to long, length in profile 1.00–1.18 times its maximum width; in female, clypeus moderately emarginated apically	*Symmorphus (Symmorphus) bifasciatus* (Linnaeus)
–	Dorsal mesepisternum punctate-puncticulate to foveolate-puncticulate, with major punctures small to moderate and sparsely spaced; in male, antennal segment 13 short to long, length in profile 0.60–1.15 times its maximum width; in female, clypeus shallowly to moderately emarginated apically	18
18	Metasomal tergum 2 nearly uniformly foveolate-puncticulate, with major punctures slightly larger and more closely spaced toward base; mesosoma at least with yellow dorsal pronotal spot or band	*Symmorphus (Symmorphus) sublaevis* (Kostylev), new record
–	Metasomal tergum 2 foveolate-puncticulate basally to punctuate-puncticulate apically, with major punctures indistinct on apical half; mesosoma black	*Symmorphus (Symmorphus) lucens* (Kostylev), new record

## Supplementary Material

XML Treatment for Symmorphus

XML Treatment for Symmorphus (Symmorphus) tianchiensis

XML Treatment for Symmorphus (Symmorphus) cavatus

XML Treatment for Symmorphus (Symmorphus) nigriclypeus

XML Treatment for Symmorphus (Symmorphus) ambotretus

XML Treatment for Symmorphus (Symmorphus) angustatus

XML Treatment for Symmorphus (Symmorphus) apiciornatus

XML Treatment for Symmorphus (Symmorphus) aurantiopictus

XML Treatment for Symmorphus (Symmorphus) bifasciatus

XML Treatment for Symmorphus (Symmorphus) foveolatus

XML Treatment for Symmorphus (Symmorphus) fuscipes

XML Treatment for Symmorphus (Symmorphus) hoozanensis

XML Treatment for Symmorphus (Symmorphus) lucens

XML Treatment for Symmorphus (Symmorphus) mizuhonis

XML Treatment for Symmorphus (Symmorphus) ornatus

XML Treatment for Symmorphus (Symmorphus) sichuanensis

XML Treatment for Symmorphus (Symmorphus) sublaevis

XML Treatment for Symmorphus (Symmorphus) violaceipennis

XML Treatment for Symmorphus (Symmorphus) yananensis

XML Treatment for Symmorphus (Symmorphus) yunnanensis
